# Realization of quantum secure direct communication over 100 km fiber with time-bin and phase quantum states

**DOI:** 10.1038/s41377-022-00769-w

**Published:** 2022-04-06

**Authors:** Haoran Zhang, Zhen Sun, Ruoyang Qi, Liuguo Yin, Gui-Lu Long, Jianhua Lu

**Affiliations:** 1 State Key Laboratory of Low-Dimensional Quantum Physics and Department of Physics, Tsinghua University, Beijing, China; 2 Beijing Academy of Quantum Information Science, Beijing, China; 3 School of Information and Technology, Tsinghua University, Beijing, China; 4 Beijing Institute of Spacecraft System Engineering, Beijing, China; 5 Frontier Science Center for Quantum Information, Beijing, China; 6 Beijing National Research Center for Information Science and Technology, Beijing, China

**Keywords:** Quantum optics, Quantum optics

## Abstract

Rapid progress has been made in quantum secure direct communication in recent years. For practical application, it is important to improve the performances, such as the secure information rate and the communication distance. In this paper, we report an elaborate physical system design and protocol with much enhanced performance. This design increased the secrecy capacity greatly by achieving an ultra-low quantum bit error rate of <0.1%, one order of magnitude smaller than that of existing systems. Compared to previous systems, the proposed scheme uses photonic time-bin and phase states, operating at 50 MHz of repetition rate, which can be easily upgraded to over 1 GHz using current on-the-shelf technology. The results of our experimentation demonstrate that the proposed system can tolerate more channel loss, from 5.1 dB, which is about 28.3 km in fiber in the previous scheme, to 18.4 dB, which corresponds to fiber length of 102.2 km. Thus, the experiment shows that intercity quantum secure direct communication through fiber is feasible with present-day technology.

## Introduction

Confidentiality of message is essential in modern communication. Traditional secure communication is using encryption, based on the computational difficulty of certain mathematical problems such as factorization large integers^1^. The rapid progress of quantum computing^2–4^ causes anxiety over the security of those traditional communication. Physical layer security based on information theory and coding is a unique way of achieving secure communications from a more fundamental level, using Wyner’s wiretap channel model^5^. Quantum secure direct communication (QSDC) is capable of estimating the secrecy capacity of realistic quantum channels enabled by the principles of quantum physics. QSDC has attracted much attention, and has become one of the strongest candidates for secure communication in the future^6^.

QSDC is different from quantum key distribution (QKD)^7,8^, which negotiates a secure key using quantum technology. QSDC and QKD perform different tasks. QSDC securely and reliably transmits information through a quantum channel with both noise and eavesdropping. Compared to classical communication where reliable transmission of information over a noisy channel is concerned, QSDC has the additional capability to ensure its security using the properties of quantum information carriers^9^. Since QSDC is a kind of communication, it is flexible to construct networks using techniques such as packet switching and so on. It has great potential for 6 G wireless communication as well^6^.

The first QSDC protocol was proposed by Long and Liu in the new millennium^10^. Since its foundation, many QSDC protocols have been proposed and experimentally demonstrated^11–20^. Recently, remarkable progress has been made, and major obstacles in its practical application have been overcome. Quantitatively security analysis and coding scheme in high-loss quantum channel have been proposed and experimentally demonstrated^21^. In that work, Qi et al. conceived a coding scheme using concatenation of low-density parity-check (LDPC) codes, pseudorandom sequences, and universal hashing families (UHF)^22,23^. This QSDC system is based on the DL04 single photon protocol^12^ with phase states. It achieved an average secure information rate of 50 bps through 1.5 km fiber. Its recent update achieves 4 kbps through fiber^24^. Meanwhile, Sun et al. proposed the idea of quantum-memory-free (QMF) protocol and designed the QMF-DL04 QSDC^12,25^. This QSDC system has dispensed of quantum memory, and significantly increased the secure information rate as well. Explicitly, it realized secure transmission at 3.2 bps through an 18 km fiber at a clock-rate of 1 MHz. The system has already transmitted message over a meaningful distance, but there are still badly need for improvement in tolerable communication loss, for support of a higher clock-rate and more effective coding scheme to approach the secrecy capacity.

Against this background, we proposed a QSDC system with a new physical system design and a new efficient coding scheme, and experimentally demonstrated the system. Specifically, the primary contributions of this work are: (1) We proposed a novel design of physical system with a new protocol. We use both photonic time-bin and phase states, and choose the time-bin states for eavesdropping detection and use the phase states for communicating the message. The system is free from phase and polarization drift, does not use the complicated active compensation subsystem. This enables an ultra-low quantum bit error rate (QBER) and the long-term stability against environmental noises. The new optical design uses a two-way structure, and it allows the returned pulses to bypass the modulators, which supports high clock-rate modulation up to 1 GHz, hence giving a high transmission rate; (2) We designed a QMF QSDC scheme using *low-density Bose-Chaudhuri-Hocquenghem* (LDBCH) codes^26–28^; (3) We implemented the system and tested it with a clock-rate of 50 MHz through fiber at different distance. The results show that the system can resist extremely great loss. In particular, the system can communicate at 22.4 kbps through about 30 km commercial fiber and 0.54 bps through 100 km ultra-low loss fiber.

## Results

### The protocol

**Fig. 1:**
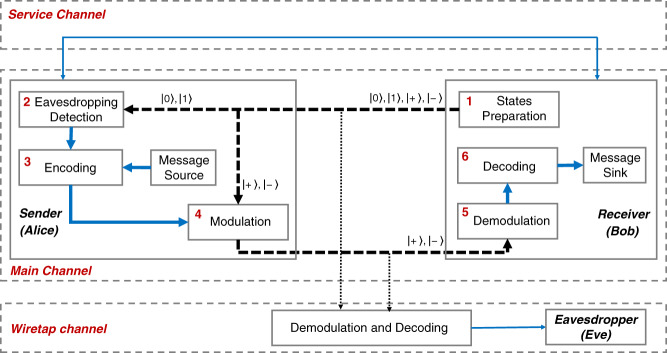
Illustration of the proposed protocol. The main channel and wiretap channel are discrete and memoryless. The service channel is a classical authenticated noiseless public channel, which transmits only the associated auxiliary data. The blue lines are classical, while qubits are transmitted over the black dotted lines

Similar to protocols in Refs. ^12,21,25^, this protocol is based on non-orthogonal states. Specifically, the time-bin states in *Z*: $\{ |0\rangle ,|1\rangle \}$ basis are used for eavesdropping detection and the phase states in *X*: $\{ | + \rangle ,| - \rangle \}$ basis are used for message delivery. The protocol is illustrated in Fig. 1. The basic steps of the protocol are:

1. *States Preparation:* Bob prepares a sequence of time-bin and phase states, $\left| 0 \right\rangle$, $\left| 1 \right\rangle$, $| + \rangle$, $| - \rangle$. States in *Z* basis, $\left| 0 \right\rangle$ and $\left| 1 \right\rangle$, represent the states passing through the short and long path of an asymmetric Mach-Zehnder interferometer, respectively. States in *X* basis, $| + \rangle$ and $| - \rangle$, are superposition states of $\left| 0 \right\rangle$ and $\left| 1 \right\rangle$. The $\left| 0 \right\rangle$ and $\left| 1 \right\rangle$ states are prepared with probability $p_z$, and the $| + \rangle$ and $| - \rangle$ states are prepared with probability $p_x$, respectively. $p_x + p_z = 1$. Then Bob sends the sequence of states to Alice over the quantum channel.
2. *Eavesdropping Detection:* After receiving the sequence of states, Alice randomly chooses some of them and measures those states in *Z* basis. Alice announces the result through the service channel. Bob compares the result with his preparations of the states in *Z* basis. Then Bob estimates the parameters of forward channel, such as QBER and loss, and tells the results to Alice through the service channel.
3. *Encoding:* According to the parameters of forward channel and the estimated parameters of the backwards channel from the previous frame, Alice encodes the message. We have conceived a variant of QMF scheme and corresponding encoding methods to dispense with quantum memory during the eavesdropping detection. Please refer to materials and methods section for more detail of encoding method.
4. *Modulation:* According to the encoded sequence, Alice operates those remain states with two unitary operations ${{{\mathbf{I}}}} = \left| 0 \right\rangle \langle 0| + \left| 1 \right\rangle \langle 1|$ and ${{{\mathbf{\sigma }}}}_{{{\mathbf{Z}}}} = \left| 0 \right\rangle \langle 0| - \left| 1 \right\rangle \langle 1|$ corresponding to bits ‘0’ and ‘1’, respectively. Next, Alice sends the modulated states to Bob.
5. *Demodulation:* Bob measures the received states that he prepared in *X* basis. Bob can obtain the sequence of operations *I* and $\sigma _Z$ corresponding to bits ‘0’ and ‘1’ by comparing the initial states and measurement results.
6. *Decoding:* Bob can decode the message successfully if the codes used in step 3 can completely remove the impact from QBER and loss. Then, Alice and Bob can repeat these steps again to transmit more information in the next frame. If Bob fails to decode the message, they can adjust the coding parameters to the appropriate. But when the channel is too noisy to convey information securely, they terminate the process. Please refer to materials and methods section for more detail of decoding method.

### Security analysis

According to wiretap channel theory^29–31^, the secrecy capacity $C_s$ of the proposed protocol is

1 $$C_s = \max \{ I(A:B) - I(A:E)\}$$

and the secure information rate $R_s$ is

2 $$R_s = R - I(A:E) \le C_s$$

where $I(A:B)$ and $I(A:E)$ are the mutual information between Alice and Bob, and between Alice and Eve respectively, *R* represents the error correction rate.

Normally, the channel between Alice and Bob can be assumed as a cascaded channel consisting of a binary symmetric channel (BSC) and a binary erasure channel (BEC) concatenated in series^21,25^. According to noisy-channel coding theorem, $I(A:B)$ cannot break Shannon limit,

3 $$I(A:B) \le Q_{Bob} \cdot [1 - h(e_x)]$$

where $Q_{Bob}$ is the total gain at Bob, $e_x$ is the QBER of received states in *X* basis, $h(x) = - xlog_2x - (1 - x)log_2(1 - x)$. represents the binary Shannon entropy.

We consider the case of collective attack^31,32^, where Eve attaches her ancillary state $\left| E \right\rangle$ to the state that Bob prepares and performs a unitary operation *U*. The initial state can be described as a density matrix $\rho ^B$,

4 $$\rho ^B = \frac{{\left| 0 \right\rangle \langle 0| + \left| 1 \right\rangle \langle 1|}}{2} = \frac{{\left| + \right\rangle \langle + | + \left| - \right\rangle \langle - |}}{2}$$

when Alice receives the states which has been disturbed by Eve, its density matrix becomes

5 $$\rho ^{BE} = U\left( {\rho ^B \otimes \left| E \right\rangle \langle E|} \right)U^ +$$

After Alice encodes the state, it becomes

6 $$\rho ^{ABE} = \frac{{\left| 0 \right\rangle _A\langle 0|_A \otimes \rho _0^{BE} + \left| 1 \right\rangle _A\langle 1|_A \otimes \rho _1^{BE}}}{2}$$

where $\left| 0 \right\rangle _A$ and $\left| 1 \right\rangle _A$ are classical bits ‘0’ and ‘1’ that Alice encodes, so that $\rho _0^{BE} = I\rho ^{BE}I^ +$ and $\rho _1^{BE} = \sigma _Z\rho ^{BE}\sigma _Z^ +$ represent the states carrying the bits ‘0’ and ‘1’, respectively. We assume the probability of encoding bits ‘0’ and ‘1’ is roughly equal. For a single photon, the mutual information between Eve and Alice’ classical bit is

7 $$\begin{array}{c}I_1 = \mathop {{\max }}\limits_{U,\left| E \right\rangle } \left\{ {S({{{\mathrm{tr}}}}_{BE}\rho ^{ABE}) + S({{{\mathrm{tr}}}}_A\rho ^{ABE}) - S(\rho ^{ABE})} \right\}\\ = \mathop {{\max }}\limits_{U,\left| E \right\rangle } \left\{ {h\left( {\frac{1}{2}} \right) + S\left( {\frac{{\rho _0^{BE} + \rho _1^{BE}}}{2}} \right) - \left[ {h\left( {\frac{1}{2}} \right) + \frac{{S(\rho _0^{BE}) + S(\rho _1^{BE})}}{2}} \right]} \right\}\\ = \mathop {{\max }}\limits_{U,\left| E \right\rangle } \left\{ {S\left( {\frac{{\rho _0^{BE} + \rho _1^{BE}}}{2}} \right) - \frac{{S(\rho _0^{BE}) + S(\rho _1^{BE})}}{2}} \right\}\end{array}$$

where $S(\rho )$ represent the von Neumann entropy. Using the Gram matrix method^21,33,34^, we obtain

8 $$\mathop {{\max }}\limits_{U,\left| E \right\rangle } \left\{ {S\left( {\frac{{\rho _0^{BE} + \rho _1^{BE}}}{2}} \right) - \frac{{S(\rho _0^{BE}) + S(\rho _1^{BE})}}{2}} \right\} = h(e_z)$$

where $e_z$ is the QBER of states in *Z* basis. Thus, we get $I(A:E)$,

9 $$I(A:E) = Q^{Eve} \cdot h(e_z)$$

where $Q^{Eve}$ is the is the maximum gain at which Eve can access the qubits. Similarly, we get,

10 $$C_s = Q^{Bob} \cdot [1 - h(e_x) - g \cdot h(e_z)]$$

where *g* represents the gap between $Q^{Eve}$ and $Q^{Bob}$.

### System implementation

**Fig. 2:**
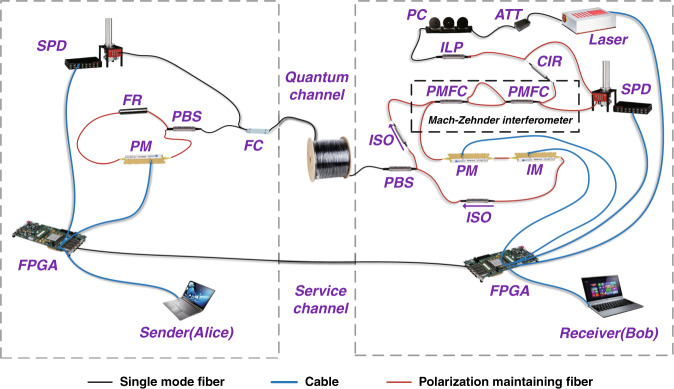
Experiment setup. Laser: 1550 nm with pulse-repetition frequency 50 MHz; FPGA field programmable gate array, ATT attenuator, PC polarization controller, ILP in-line polarizer, CIR optical circulator, PBS polarization beam splitter, FC 90:10 filter coupler, PMFC polarization maintaining filter coupler, PM phase modulator, IM intensity modulator with extinction ratio of 45.1 dB, ISO isolator, FR 90 degree Faraday rotator, SPD superconducting nanowire single-photon detector with over 85% detection efficiency, 50 Hz dark count rate and 15 ns reset time. The asymmetric Mach-Zehnder interferometer consists of two PMFC, and the delay length is about 2 m

The experimental setup is shown in Fig. 2. Bob uses an asymmetric Mach-Zehnder interferometer to split one pulse into two sub-pulses. The relative phases and intensity of sub-pulses are modulated by a phase modulator and an intensity modulator respectively. For the information carrier states $| + \rangle$ and $| - \rangle$, the birefringence drift is auto-compensated by the two-way structure with a Faraday rotator. These sub-pulses are generated and demodulated accurately by the same asymmetric Mach-Zehnder interferometer at Bob’s end. This plug-and-play design also allows the returned pulses to bypass the modulators, which can support much higher clock-rate over 1 GHz for modulation^34^. For the states $|0\rangle$ and $|1\rangle$ for eavesdropping detection, the time of arrival at Alice’ end could be directly detected by a single-photon detector. Thus, the whole system is robust for both polarization and phase without active feedback, and without a pair of matching interferometers, which greatly increases the reliability of the system with an ultra-low QBER.

The experimental parameters and performances of the system are shown in Table 1 at typical distance of 30 km and 100 km. We adjusted the channel loss to 6 dB using 25.2 km fiber and attenuator, which represents 30 km commercial fiber. Then, we replaced them with a 100 km ultra-low loss fiber to estimate the longest communication distance. For simplification and optimization, we consider the case of infinite length code. The transmittance at Alice’s end is $\eta ^{Alice}$, and the efficiency of demodulation at Bob’s end is $\eta ^{Bob}$.

**Table 1:** Experimental parameters and performance

Distance	Channel loss	Mean photon number	*η*^*Alice*^	*η*^*Bob*^	*e*_*x*_	*e*_*z*_	*R*_*s*_
30 km	6 dB	0.1/pulse	0.398	0.275	0.001	0.0003	22.4 kbps
100 km	15.8 dB	0.1/pulse	0.398	0.275	0.025	0.0004	0.54 bps

### Comparisons of secrecy capacity and secure information rate

**Fig. 3:**
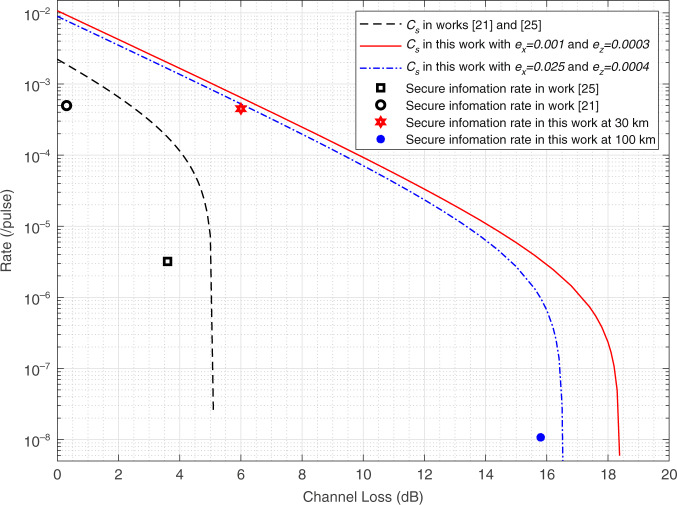
Simulation with experimental parameters. Comparison of secrecy capacity and secure information rate between previous works and this work.

Here, we compare the secrecy capacity and secure information rate of the proposed QSDC system with previous works^21,25^, which is shown in Fig. 3. The red curve and blue curve are the evaluated secrecy capacity using the parameters at distance of 30 and 100 km, respectively. The red and blue dots are the secure information rate at distance of 30 and 100 km, respectively. The details of coding method will be described in materials and methods section.

Although this plug-and-play design without active feedback eliminates the mismatching and phase drift of interferometer^34^, the QBER, especially in *X* basis, becomes larger as the distance gets longer due to the Rayleigh backscattering. Since the system operates at a repetition rate of 50 MHz, the backscattering photons are evenly distributed over the entire time domain. Therefore, the use of dispersion compensating fiber at Bob’ end can reduce the count of backscattering photons, which realized our system to transmit the secure information over 100 km. We assume that the QBER with different channel loss is approximately between the QBER in the above two situations, so the actual secrecy capacity curve will be between the red curve and blue curve. Some fiber manufacturing processes, lower repetition rate or time division method can further reduce the impact of Rayleigh backscattering^34^, which can make actual secrecy capacity approach the red line at long distance. From Fig. 3, it can be seen clearly that a huge improvement of performance of this work over the results in previous works^21,25^ is obtained. For a detailed comparison, some specific results are shown in Table 2. The secrecy capacity is much larger than the previous works under the same channel loss, especially in the high channel loss parts. Meanwhile, the maximum tolerable communication loss is improved from 5.1 to 18.4 dB, which is over 100 km when using low loss fiber of $0.18\,dB/km$.

**Table 2:** Evaluation results of secrecy capacity with several typical channel loss

Channel loss	0 dB	2.0 dB	5.1 dB	10 dB	14 dB	18 dB
Works^20,24^	2.2 × 10^−3^	6.5 × 10^−4^	2.4 × 10^−8^	0	0	0
This work	1.1 × 10^−2^	4.2 × 10^−3^	9.9 × 10^−4^	9.3 × 10^–5^	1.1 × 10^–5^	2.3 × 10^–7^

## Discussion

In this work, a QMF QSDC with photonic time-bin and phase quantum states is proposed. We designed a new physical system and constructed the QSDC protocol. The QBER and instability are improved to an ultra-low level due to the intrinsic properties of the system. Then, a variant of QMF QSDC was constructed using the secure coding, it dispenses the use of quantum memory. Finally, we implemented the scheme, conducted tests at distance of 30 km and 100 km, and compared the results with previous works.

Consequently, this system has a much higher clock-rate and maximum tolerable channel loss. Meanwhile, a new coding method using masking can further improve the performance of the system^35^. We realized the high-performance QSDC system, which is promising for the use in future intercity communication.

## Materials and methods

### Coding method for quantum-memory-free QSDC

In this section, we introduce the QMF QSDC protocol used in our system. It is a variant of the QMF-DL04 QSDC protocol, conforming to the general idea of QMF QSDC^25^. The main difference is that the joint encryption and error-control (JEEC) coding is replaced by the secure coding based on UHF^21,22^. Meanwhile, the *generalized LDPC code based on the Hadamard codes and repetition* (GLHR) codes in QMF-DL04 QSDC is replaced by the *LDBCH concatenated with repetition* (LDBCHR) codes to enhance the reliability of the communication. All the vectors in the rest of this paper are row vectors, unless stated otherwise.

#### Protocol structure

**Fig. 4:**
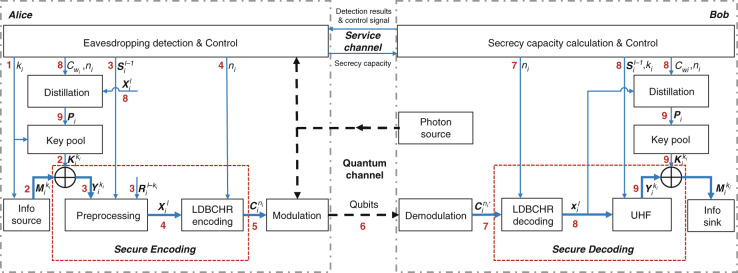
Structure of our variant of QMF QSDC protocol and the data stream of the *i*-th frame. The black dotted lines carry qubits, and the blue lines carry classical bits. The rectangular boxes are the modules while the directed lines between them represent the data stream. Besides, the symbols near the lines are the data in the stream

Figure 4 details how Alice sends the *i*-th frame of message to Bob over the quantum channel with the aid of the classical service channel in our variant of QMF QSDC protocol.

The symbols’ definitions are introduced in the ascending order of the numbers beside them:

1. $k_i$ is the selected information length of the *i*-th frame.
2. ${{{\mathrm{M}}}}_{{{\mathrm{i}}}}^{{{{\mathrm{k}}}}_{{{\mathrm{i}}}}}$ is a $k_i$-bit message, which will be encoded by the secure encoding module. ${{{\mathrm{K}}}}_{{{\mathrm{i}}}}^{{{{\mathrm{k}}}}_{{{\mathrm{i}}}}}$ taken from the key pool is the key to encrypt ${{{\mathrm{M}}}}_{{{\mathrm{i}}}}^{{{{\mathrm{k}}}}_{{{\mathrm{i}}}}}$.
3. ${{{\mathrm{Y}}}}_{{{\mathrm{i}}}}^{{{{\mathrm{k}}}}_{{{\mathrm{i}}}}}$, one input of the preprocessing module, represents the ciphertext given by ${{{\mathrm{Y}}}}_{{{\mathrm{i}}}}^{{{{\mathrm{k}}}}_{{{\mathrm{i}}}}} = {{{\mathrm{ M}}}}_{{{\mathrm{i}}}}^{{{{\mathrm{k}}}}_{{{\mathrm{i}}}}} \oplus {{{\mathrm{ K}}}}_{{{\mathrm{i}}}}^{{{{\mathrm{k}}}}_{{{\mathrm{i}}}}}$. The other two inputs are a local random bit sequence ${{{\mathrm{R}}}}_{{{\mathrm{i}}}}^{{{{\mathrm{l - k}}}}_{{{\mathrm{i}}}}}$ and a random bit sequence $S_i^{l - 1}$ shared over the service channel.
4. ${{{\mathrm{X}}}}_{{{\mathrm{i}}}}^{{{\mathrm{l}}}}$ is the output of the preprocessing and an input of the LDBCHR ($n_i$, *l*).
5. ${{{\mathrm{C}}}}_{{{\mathrm{i}}}}^{{{{\mathrm{n}}}}_{{{\mathrm{i}}}}}$ is the codeword to be modulated on qubits using the method in Section II.
6. Qubits are transmitted to Bob through the quantum channel.
7. ${{{\mathrm{C}}}}_{{{\mathrm{i}}}}^{{{{\mathrm{n}}}}_{{{\mathrm{i}}}}\prime }$ is the received codeword and an input of LDBCHR decoding module.
8. After the transmission of ${{{\mathrm{C}}}}_{{{\mathrm{i}}}}^{{{{\mathrm{n}}}}_{{{\mathrm{i}}}}}$, Alice and Bob can obtain the $I_i(A:B)$, $I_i(A:E)$ and $C_{s_i}$ of the *i*-th frame. If the decoding of LDBCH code is correct, ${{{\mathrm{X}}}}_{{{\mathrm{i}}}}^{{{\mathrm{l}}}}$ can further be used as an input of the distillation module and the UHF module.
9. Alice and Bob can distill a same secret key ${{{\mathrm{P}}}}_{{{\mathrm{i}}}}$ from ${{{\mathrm{X}}}}_{{{\mathrm{i}}}}^{{{\mathrm{l}}}}$ according to the results of eavesdropping detection and secrecy capacity calculation, also making use of UHF^21,22^. ${{{\mathrm{P}}}}_{{{\mathrm{i}}}}$ is stored in a first-in first-out (FIFO) pool.

#### Work flow

The quantum processes of delivering ${{{\mathrm{C}}}}_{{{\mathrm{i}}}}^{{{{\mathrm{n}}}}_{{{\mathrm{i}}}}}$ through the quantum channel and estimating $I_i(A:E)$ are the same as those of the proposed scheme in results section. Here we only concentrate on the classical data stream. The details of the preprocessing, LDBCH encoding/decoding and UHF modules will be clarified in the following.

Note that $I_i(A:E)$ cannot be acquired until the *i*-th ${{{\mathrm{C}}}}_{{{\mathrm{i}}}}^{{{{\mathrm{n}}}}_{{{\mathrm{i}}}}}$ has been transmitted. In fact, this is exactly why the previous QSDC protocols relied on quantum memory. Hence, if Alice wants to communicate the *i*-th frame to Bob, she has to determine $k_i$ and $n_i$ first, which should satisfy following equations:

11 $$\frac{{k_i}}{{n_i}} \le \frac{l}{{n_i}} - I_{i - 1}(A:E)$$

12 $$\frac{l}{{n_i}} \le I_{i - 1}(A:B)$$

in which ($I_i(A:B)$, $I_i(A:E)$) of the *i*-th frame are predicted by those of the ($i - 1$)-th frame. Equation (12) is for the communication reliability^36^, while Eq. (11) is the requirement of security^22,25^.

During the whole communication process, the system operates in two different states depending on whether there is enough secret key (no shorter than $k_i$ in Eq. (11)) for the transmission of *i*-th information frame:

- ***Preparing State:*** *There is insufficient key to encrypt* ${{{\mathrm{M}}}}_{{{\mathrm{i}}}}^{{{{\mathrm{k}}}}_{{{\mathrm{i}}}}}$. Information source and preprocessing modules do not work. Meanwhile, ${{{\mathrm{X}}}}_{{{\mathrm{i}}}}^{{{\mathrm{l}}}}$ is a random bit sequence produced by a random number generator^37,38^. If Bob flawlessly receives the random ${{{\mathrm{X}}}}_{{{\mathrm{i}}}}^{{{\mathrm{l}}}}$, from which Alice and Bob can distill a common key ${{{\mathrm{P}}}}_{{{\mathrm{i}}}}$ whose length is $n_i \cdot [R_i - I_i(A:E)]$, where $R_i = l/n_i$. Otherwise, if Bob fails decoding the codeword, Alice have to send another random bit sequence. The workflow of this state is summarized as follows:

1. Alice generates a sequence of random bits ${{{\mathrm{X}}}}_{{{\mathrm{i}}}}^{{{\mathrm{l}}}}$.
2. ${{{\mathrm{X}}}}_{{{\mathrm{i}}}}^{{{\mathrm{l}}}}$ is encoded to ${{{\mathrm{C}}}}_{{{\mathrm{i}}}}^{{{{\mathrm{n}}}}_{{{\mathrm{i}}}}}$ by the LDBCHR encoder, where $n_i$ satisfies Eq. (12).
3. Alice sends ${{{\mathrm{C}}}}_{{{\mathrm{i}}}}^{{{{\mathrm{n}}}}_{{{\mathrm{i}}}}}$ to Bob.
4. Bob receives ${{{\mathrm{C}}}}_{{{\mathrm{i}}}}^{{{{\mathrm{n}}}}_{{{\mathrm{i}}}}\prime }$ from the quantum channel.
5. If the decoding of ${{{\mathrm{C}}}}_{{{\mathrm{i}}}}^{{{{\mathrm{n}}}}_{{{\mathrm{i}}}}\prime }$ is incorrect, return to step 2. If not, Alice and Bob distill ${{{\mathrm{P}}}}_{{{\mathrm{i}}}}$ from ${{{\mathrm{X}}}}_{{{\mathrm{i}}}}^{{{\mathrm{l}}}}$ according to $R_i = l/n_i$ and $I_i(A:E)$.

Loop through above 5 steps until the key is long enough to encrypt ${{{\mathrm{M}}}}_{{{\mathrm{i}}}}^{{{{\mathrm{k}}}}_{{{\mathrm{i}}}}}$ and change to the communication state.

- ***Communication state:*** *The system has enough key to encrypt* ${{{\mathrm{M}}}}_{{{\mathrm{i}}}}^{{{{\mathrm{k}}}}_{{{\mathrm{i}}}}}$: All modules works. If ${{{\mathrm{X}}}}_{{{\mathrm{i}}}}^{{{\mathrm{l}}}}$ is successfully recovered by the LDBCHR encoder, Bob can get the message ${{{\mathrm{M}}}}_{{{\mathrm{i}}}}^{{{{\mathrm{k}}}}_{{{\mathrm{i}}}}}$ included in the *i*-th frame. Alice and Bob can also distill a common ${{{\mathrm{P}}}}_{{{\mathrm{i}}}}$ from ${{{\mathrm{X}}}}_{{{\mathrm{i}}}}^{{{\mathrm{l}}}}$. Or else, if the decoding fails, Alice has to re-transmit the corresponding message by a new key from the pool. Similarly, the workflow of this state is summarized as follows:

1. Information source outputs a message ${{{\mathrm{M}}}}_{{{\mathrm{i}}}}^{{{{\mathrm{k}}}}_{{{\mathrm{i}}}}}$, whose length $k_i$ satisfies Eq. (11).
2. XOR module encrypts ${{{\mathrm{M}}}}_{{{\mathrm{i}}}}^{{{{\mathrm{k}}}}_{{{\mathrm{i}}}}}$: ${{{\mathrm{Y}}}}_{{{\mathrm{i}}}}^{{{{\mathrm{k}}}}_{{{\mathrm{i}}}}} = {{{\mathrm{ M}}}}_{{{\mathrm{i}}}}^{{{{\mathrm{k}}}}_{{{\mathrm{i}}}}} \oplus {{{\mathrm{ K}}}}_{{{\mathrm{i}}}}^{{{{\mathrm{k}}}}_{{{\mathrm{i}}}}}$, where ${{{\mathrm{K}}}}_{{{\mathrm{i}}}}^{{{{\mathrm{k}}}}_{{{\mathrm{i}}}}}$ is from the key pool.
3. Preprocessing: $\left( {{{{\mathrm{Y}}}}_{{{\mathrm{i}}}}^{{{{\mathrm{k}}}}_{{{\mathrm{i}}}}}{{{\mathrm{,S}}}}_{{{\mathrm{i}}}}^{{{{\mathrm{l}}}} - {{{\mathrm{1}}}}}{{{\mathrm{,R}}}}_{{{\mathrm{i}}}}^{{{{\mathrm{l}}}} - {{{\mathrm{k}}}}_{{{\mathrm{i}}}}}} \right)\mathop{\longrightarrow}\limits^{{f^{ - 1}}}{{{\mathrm{X}}}}_{{{\mathrm{i}}}}^{{{\mathrm{l}}}}$, where we denote the preprocessing process as $f^{ - 1}$.
4. Encode ${{{\mathrm{X}}}}_{{{\mathrm{i}}}}^{{{\mathrm{l}}}}$ to get the codeword ${{{\mathrm{C}}}}_{{{\mathrm{i}}}}^{{{{\mathrm{n}}}}_{{{\mathrm{i}}}}}$.
5. Modulate ${{{\mathrm{C}}}}_{{{\mathrm{i}}}}^{{{{\mathrm{n}}}}_{{{\mathrm{i}}}}}$ onto the qubits.
6. Alice sends the modulated qubits to Bob. Bob demodulates them and receives ${{{\mathrm{C}}}}_{{{\mathrm{i}}}}^{{{{\mathrm{n}}}}_{{{\mathrm{i}}}}\prime }$.
7. The LDBCHR decoder try to recover ${{{\mathrm{X}}}}_{{{\mathrm{i}}}}^{{{\mathrm{l}}}}$. If it succeeds, continue to the next step. In case of a failure, return to step 2.
8. Universal hashing: $({{{\mathrm{X}}}}_{{{\mathrm{i}}}}^{{{\mathrm{l}}}}{{{\mathrm{,S}}}}_{{{\mathrm{i}}}}^{{{{\mathrm{l - 1}}}}})\mathop{\longrightarrow}\limits^{f}{{{\mathrm{Y}}}}_{{{\mathrm{i}}}}^{{{{\mathrm{k}}}}_{{{\mathrm{i}}}}}$, where *f* represents the universal hashing process. Meanwhile, Alice and Bob distill a key ${{{\mathrm{P}}}}_{{{\mathrm{i}}}}$ from ${{{\mathrm{X}}}}_{{{\mathrm{i}}}}^{{{\mathrm{l}}}}$.
9. Bob uses ${{{\mathrm{K}}}}_{{{\mathrm{i}}}}^{{{{\mathrm{k}}}}_{{{\mathrm{i}}}}}$to decrypt ${{{\mathrm{Y}}}}_{{{\mathrm{i}}}}^{{{{\mathrm{k}}}}_{{{\mathrm{i}}}}}$: ${{{\mathrm{M}}}}_{{{\mathrm{i}}}}^{{{{\mathrm{k}}}}_{{{\mathrm{i}}}}} = {{{\mathrm{ Y}}}}_{{{\mathrm{i}}}}^{{{{\mathrm{k}}}}_{{{\mathrm{i}}}}} \oplus {{{\mathrm{ K}}}}_{{{\mathrm{i}}}}^{{{{\mathrm{k}}}}_{{{\mathrm{i}}}}}$. Meanwhile, Alice and Bob put ${{{\mathrm{P}}}}_{{{\mathrm{i}}}}$ into the pool for later use.

Loop through these steps until the key is insufficient or all messages have been successfully transmitted. Then, the system changes to the preparing state or finish the communication process, respectively.

#### Secure coding scheme

**Fig. 5:**
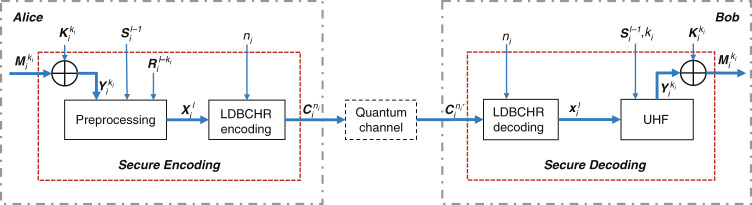
Secure coding. Structure of the secure coding scheme and the data stream of the *i*th frame, which consists of the XOR, preprocessing, LDBCHR encoding/decoding and UHF modules.

Figure 5 represents the structure of the secure coding scheme. The XOR module perform the bit-by-bit exclusive or operations. The details of other modules are as follows. Note that the subscript *i* is omitted in the following of this part.

- ***Preprocessing***, $({{{\mathrm{Y}}}}^{{{\mathrm{k}}}}{{{\mathrm{,S}}}}^{{{{\mathrm{l}}}} - {{{\mathrm{1}}}}}{{{\mathrm{,R}}}}^{{{{\mathrm{l}}}} - {{{\mathrm{k}}}}})\mathop{\longrightarrow}\limits^{{f^{ - 1}}}{{{\mathrm{X}}}}^{{{\mathrm{l}}}}.{{{\mathrm{Y}}}}^{{{\mathrm{k}}}}$ is the input encrypted bit sequence. ${{{\mathrm{S}}}}^{{{{\mathrm{l}}}} - {{{\mathrm{1}}}}} = (s_1,s_2, \cdots ,s_{l - 1})$ is a shared random bit sequence, which is used to construct the Toeplitz matrix ${{{\mathbf{T}}}}^{(l - k) \times k}$:
  13 $${{{\mathbf{T}}}}^{(l - k) \times k} = \left[ {\begin{array}{*{20}{c}} {s_{l - k}} & {s_{l - k + 1}} & \cdots & {s_{l - 1}} \\ {s_{l - k - 1}} & {s_{l - k}} & \cdots & {s_{l - 2}} \\ \vdots & \vdots & \ddots & \vdots \\ {s_1} & {s_2} & \cdots & {s_k} \end{array}} \right]$$
  Another input ${{{\mathrm{R}}}}^{{{{\mathrm{l}}}} - {{{\mathrm{k}}}}} = (r_1,\;r_2, \cdots ,\;r_{l - k})$ is a local random bit sequence. Then, the output ${{{\mathrm{X}}}}^{{{\mathrm{l}}}}$ of preprocessing is ${{{\mathrm{X}}}}^{{{\mathrm{l}}}} = \left[ {{{{\mathrm{R}}}}^{{{{\mathrm{l}}}} - {{{\mathrm{k}}}}}{{{\mathrm{,}}}}\;{{{\mathrm{Y}}}}^{{{\mathrm{k}}}} - {{{\mathrm{R}}}}^{{{{\mathrm{l}}}} - {{{\mathrm{k}}}}}{{{\mathbf{T}}}}^{(l - k) \times k}} \right]$.
- ***UHF***, $({{{\mathrm{X}}}}^{{{\mathrm{l}}}}{{{\mathrm{,S}}}}^{{{{\mathrm{l}}}} - {{{\mathrm{1}}}}})\mathop{\longrightarrow}\limits^{f}{{{\mathrm{Y}}}}^{{{\mathrm{k}}}}$ The UHF module of Bob also uses ${{{\mathbf{T}}}}^{(l - k) \times k}$ to recover the ${{{\mathrm{Y}}}}^{{{\mathrm{k}}}}$:
  14 $${{{\mathrm{X}}}}^{{{\mathrm{l}}}}\left[ {\begin{array}{*{20}{c}} {{{{\mathbf{T}}}}^{(l - k) \times k}} \\ {{{{\mathbf{I}}}}^{k \times k}} \end{array}} \right] = \left[ {{{{\mathrm{R}}}}^{{{{\mathrm{l}}}} - {{{\mathrm{k}}}}}{{{\mathrm{,Y}}}}^{{{\mathrm{k}}}} - {{{\mathrm{R}}}}^{{{{\mathrm{l}}}} - {{{\mathrm{k}}}}}{{{\mathbf{T}}}}^{(l - k) \times k}} \right]\left[ {\begin{array}{*{20}{c}} {{{{\mathbf{T}}}}^{(l - k) \times k}} \\ {{{{\mathbf{I}}}}^{k \times k}} \end{array}} \right] = {{{\mathrm{Y}}}}^{{{\mathrm{k}}}}$$
- ***LDBCHR codes***, LDBCHR is a kind of cascaded codes based on LDBCH^26,28^ and repetition codes. The encoding and decoding procedures are as follows.

The input ${{{\mathrm{X}}}}^{{{\mathrm{l}}}}$ is first encoded by a $(n_1,l)$ LDBCH encoder^25,27^. Denote the output as ${{{\mathrm{C}}}}_{{{\mathrm{L}}}}^{{{{\mathrm{n}}}}_{{{\mathrm{1}}}}} = (c_{L,1},c_{L,2}, \cdots ,c_{L,n_1})$. Then, map $c_{L,i}\;(i = 1,2, \cdots ,n_1)$ to a codeword of the $(n/n_1,1)$ repetition code, ${{{\mathrm{C}}}}_{{{{\mathrm{R,i}}}}}^{{{{\mathrm{n/n}}}}_{{{\mathrm{1}}}}} = (\underbrace {c_{L,i},c_{L,i}, \cdots ,c_{L,i}}_{n/n_1{{{\mathrm{times}}}}})$. Hence, we get the codeword ${{{\mathrm{C}}}}^{{{\mathrm{n}}}} = \left( {{{{\mathrm{C}}}}_{{{{\mathrm{R,1}}}}}^{{{{\mathrm{n/n}}}}_{{{\mathrm{1}}}}}{{{\mathrm{,C}}}}_{{{{\mathrm{R,2}}}}}^{{{{\mathrm{n/n}}}}_{{{\mathrm{1}}}}}{{{\mathrm{,}}}} \cdots {{{\mathrm{,C}}}}_{{{{\mathrm{R,n}}}}_{{{\mathrm{1}}}}}^{{{{\mathrm{n/n}}}}_{{{\mathrm{1}}}}}} \right)$ of a $(n,l)$ LDBCHR code.

As for decoding, Bob first calculates the log-likelihood ratio (LLR) sequence ${{{\mathrm{U}}}}^{{{{\mathrm{n}}}}_{{{\mathrm{1}}}}}{{{\mathrm{ = }}}}(u_1,u_2, \cdots ,u_{n_1})$ of the received $\widehat {{{{\mathrm{C}}}}_{{{\mathrm{L}}}}^{{{{\mathrm{n}}}}_{{{\mathrm{1}}}}}}$. Then, input ${{{\mathrm{U}}}}^{{{{\mathrm{n}}}}_{{{\mathrm{1}}}}}$ to the decoding algorithm of LDBCH codes^25^ and Bob can recover ${{{\mathrm{X}}}}^{{{\mathrm{l}}}}$ if the decoding is correct.

#### Error rate performance

**Fig. 6:**
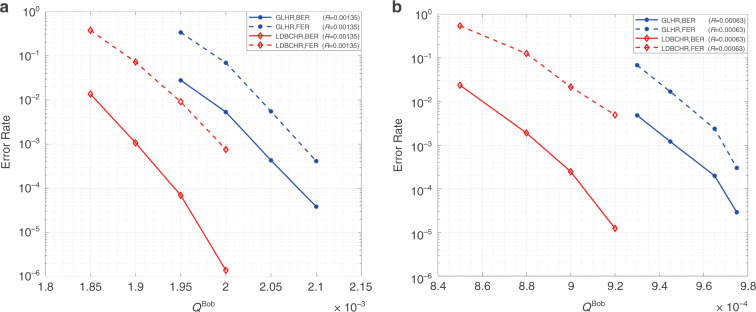
Comparison of bit error rate/frame error rate performances between the GLHR and LDBCHR. **a** *R* = 0.00135; **b** *R* = 0.00063

This evaluation compares the error rate performances of the LDBCHR codes and the GLHR codes over the cascaded BSC-BEC channel whose error rate of BSC is set to $e_x = 0.01$ (the same as ref. ^25^). All the code parameters of the GLHR codes are also consistent with that in ref. ^39^. A (4000, 2000) random Mackay LDPC code with column weight 3 and row weight 6 and the Hadamard code of order 4 are used for constructing the GLHR codes. We designed the LDBCHR codes with similar parameters. Specifically, the original LDPC for LDBCHR is a (4000, 2000) code generated by the mixed progressive edge growth - approximate cycle extrinsic message degree (PEG-ACE) algorithm^40^, whose node perspective degree distributions of variable nodes (VNs) and variable nodes (CNs) are

15 $$\left\{ {\begin{array}{*{20}{l}} {\lambda (x) = 0.5075x^1 + 0.0200x^2 + 0.4375x^3 + 0.0350x^5} \hfill \\ {\rho (x) = 0.9300x^5 + 0.0700x^6} \hfill \end{array}} \right.$$

where the maximal degrees of VNs and CNs are 6 and 7, respectively. Moreover, the (15, 5) and (31, 6) BCH code constraints are used to replace the single parity check constraints of the degree 6 CNs and degree 7 CNs, respectively^28^. The repeating times of LDBCHR and GLHR codes in Fig. 6a both are 61 while those in Fig. 6b both are 131. The overall coding rates are $R = 0.00135$ and $R = 0.00063$. We set the maximum number of iterations $I_{max} = 30$ for both the GLHR decoder and the LDBCHR decoder. As demonstrated in Fig. 6a, b, the LDBCHR designed in this work outperform GLHR codes in work^24^ on the aspects of error rate performances. Specifically, the required receiving rates of LDBCHR are about 8% lower than that of GLHR for the bit error rate after decoding around 10^−5^.
